# Toward Unified Autonomous Scattering Experiments: A Cross-Facility Case Study at ALS and PETRA III

**DOI:** 10.1021/photonsci.5c00044

**Published:** 2026-03-02

**Authors:** Wiebke Koepp, Benedikt Sochor, Dylan McReynolds, Tanny Chavez, Marcus Noack, Raja Vyshnavi Sriramoju, Aidan H. Coffey, Yunfei Wang, Enno Henn, Anna Katharina Sambale, Eric Euchler, Damon English, Frank Schlünzen, Eric Schaible, Chenhui Zhu, Sarathlal Koyiloth Vayalil, Stephan V. Roth, Alexander Hexemer

**Affiliations:** † Advanced Light Source, 606709 Lawrence Berkeley National Laboratory, Berkeley, California 94720, United States; ‡ 28332 Deutsches Elektronen-Synchrotron DESY, Hamburg 22607, Germany; § Applied Mathematics and Computational Research Division, Lawrence Berkeley National Laboratory, Berkeley, California 94720, United States; ∥ Polymer Materials Engineering, 28408 Leibniz Institute of Polymer Research Dresden, Dresden 01069, Germany; ⊥ School of Polymer Science and Engineering, 5104 The University of Southern Mississippi, Hattiesburg, Mississippi 39406, United States; # Applied Science Cluster, UPES, Dehradun, Uttarakhand 248007, India; ∇ Department of Fibre and Polymer Technology, KTH Royal Institute of Technology, Stockholm SE-10044, Sweden

**Keywords:** autonomous experiments, Gaussian process regression, small- and wide-angle scattering, cross-facility workflows, beamline automation

## Abstract

Autonomous experiments rely on the integration of control, data acquisition, analysis, and decision-making frameworks. While such systems have been demonstrated at individual facilities, adapting them to additional instruments remains challenging due to differences in local infrastructure. We present a modular workflow that connects existing open-source tools for data access (Tiled), workflow orchestration (Prefect), analysis and visualization (pyFAI, Plotly Dash), and Gaussian-process-based adaptive sampling (gpCAM) into a unified framework for autonomous scattering experiments. The same configuration operates across two synchrotron beamlines (ALS 7.3.3 and PETRA III P03) with only minimal facility-specific adjustments, as shown in proof-of-concept demonstrations. This validates that a consistent design emphasizing modularity and shared interfaces can ease deployment across diverse experimental environments. The resulting framework provides a flexible foundation for extending autonomous control and analysis capabilities beyond a single beamline or instrument.

## Graphical abstract

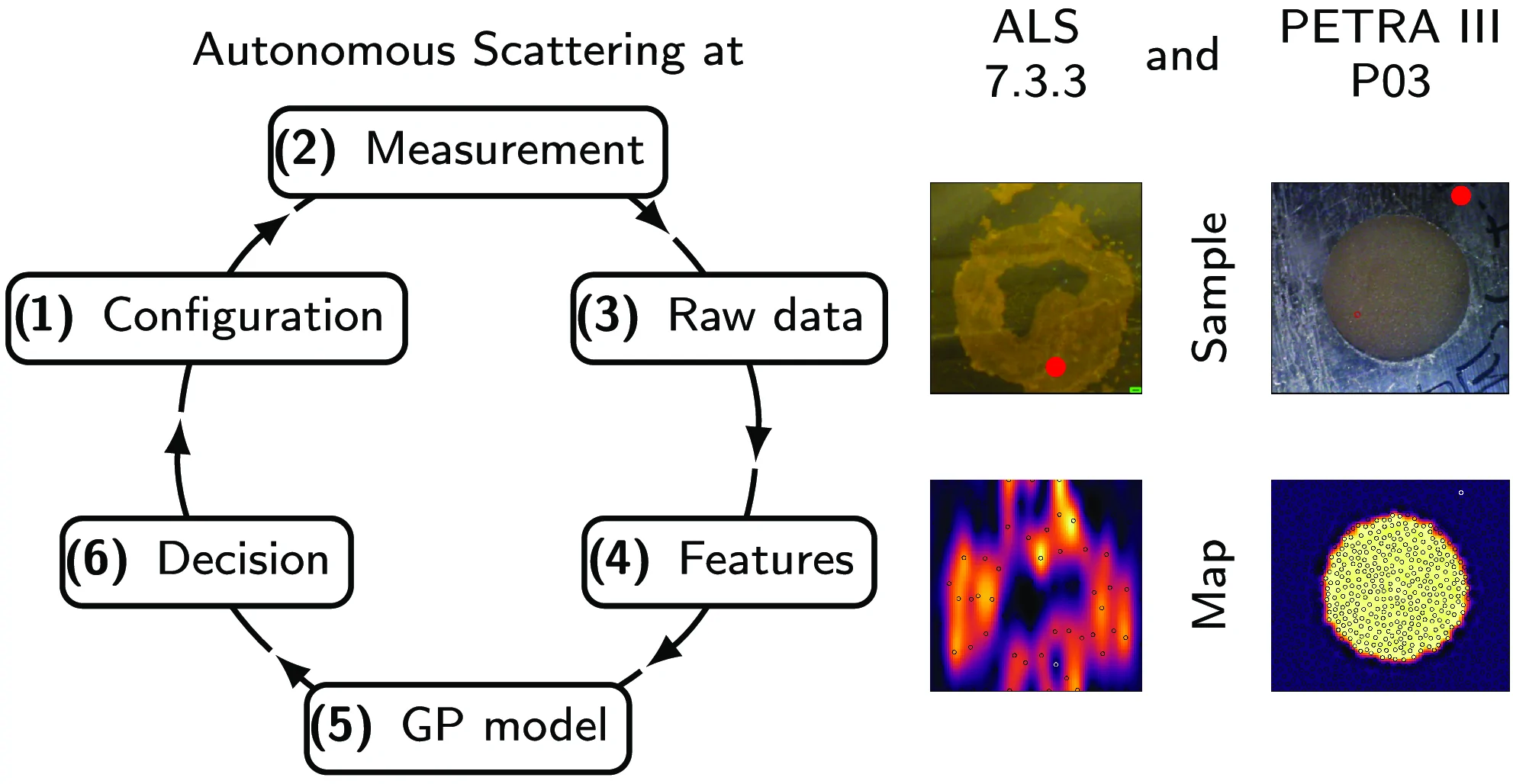

## Introduction

Autonomous experiments rely on seamless integration of control systems, data acquisition, data processing, and optimization frameworks. However, the inherent variability in facility- or beamline-specific infrastructure components poses a challenge for developing generalized experiment setups and presents obstacles for broader deployment across facilities, including cross-facility experiments.

To overcome such obstacles, the scientific community is generally moving towards autonomous experiments and self-driving labs, with modular and flexible computing architectures as key ingredients.^1,2^

Automation and real-time data workflows have also been identified as top strategic priorities by major research infrastructure programs worldwide. In the United States, the Department of Energy’s Basic Energy Sciences program emphasized autonomous control of scientific systems as a central research need across several Basic Research Needs roundtables^3–5^ on artificial intelligence, machine learning and data for scientific discovery. Similarly, the League of European Accelerator-based Photon Sources (LEAPS) Technology Roadmap^6^ highlights automation, high-throughput measurements, and AI-enabled feedback as essential for improving efficiency, reproducibility, and interoperability across European synchrotron and free-electron laser facilities. These complementary strategies underscore a shared international vision that modular, AI-integrated infrastructures are key to realizing next-generation autonomous and data-driven experiments.

For X-ray scattering experiments at synchrotron facilities, recent work has established modular autonomous experimentation loops. Yager and Noack et al. demonstrated Gaussian-process-based mapping and adaptive sampling for small- and grazing-incidence scattering, illustrating how real-time feedback can accelerate structure discovery.^7–9^ These concepts have since been extended to related scattering modalities, including diffraction, reflectometry, and formulation studies that couple synthesis and in-situ scattering under algorithmic control.^10–12^ Similar autonomous control strategies have also been demonstrated for neutron scattering^13,14^ and thin-film synthesis,^15^ underscoring the growing convergence of autonomous methodologies across photon and neutron science and the need for infrastructures that generalize across beamlines and facilities.

Autonomous experimentation is particularly relevant for materials science problems where structure formation spans multiple length scales and evolves sensitively with processing conditions. Complementary to such efforts, machine learning methods are increasingly used to extract structural information from X-ray scattering data, supporting both interpretation and automated analysis. Examples include the characterization of nanoscale domain morphology in organic photovoltaic materials,^16^ the inference of biomolecular structures in solution,^17^ and the analysis of sol-gel nanoporous materials.^18^ These advances highlight the importance of automated, reusable feature-extraction pipelines, and motivate the need for autonomous workflows that allow such ML-based methods to be integrated with ease.

In this study, we present our efforts to establish a robust infrastructure for autonomous small- and wide-angle scattering (SAXS/WAXS) experiments for materials science at two different synchrotron X-ray scattering beamlines: the Advanced Light Source (SAXS/WAXS) beamline 7.3.3 ^19^ (Berkeley, CA) and the DESY PETRA-III micro- and nano-focus SAXS/WAXS beamline (MiNaXS) beamline P03 ^20^ (Deutsches Elektronen-Synchrotron DESY, Hamburg). We detail where facility-specific adaptations are necessary, and how we leveraged modular software components to unify as many aspects of the autonomous loop as possible.

We include proof-of-concept demonstrations that validate end-to-end integration of acquisition, objective evaluation, and decision-making at both facilities. While these examples use simple objectives (e.g., uncertainty of derived features for sample mapping), the framework is general and supports broader classes of scattering-derived objectives.

Our approach focuses on modularity and standardized interfaces, enabling autonomous control, data access, and decision-making components to function cohesively across different beamline environments. This design directly addresses a key practical challenge: adapting generalized autonomous frameworks to the constraints of individual beamline and facility infrastructures.

## Results

To demonstrate autonomous SAXS and WAXS experiments across two synchrotron facilities, we prototyped a computational infrastructure that integrates beamline control, data acquisition, data handling, reduction, feature extraction, and decision-making into a unified closed-loop workflow. Our goal was to create a modular system that can be adapted to different beamlines while maintaining consistent data structures, analysis steps, and communication between components. This modularity prepares the pipeline to be readily adoptable at additional instruments, and in future contexts. This is particularly relevant in light of ongoing and planned upgrades at many synchrotron facilities worldwide that are accompanied by modernization of beamline control and data acquisition systems, and integration of additional hardware suited for high-throughput experiments, like the recent installation of a sample-exchange robot at beamline 7.3.3.^21^

In the following, we describe the computational architecture and software components, and their integration with beamline-specific control systems and computing resources. In order to show the validity of our approach for materials science, we investigate the coffee-ring effects induced by drying a salt solution—where solvent evaporation induces self-assembly and crystal formation—as well as the nanostructure of a co-extruded polymer composite, which is defined by the processing conditions.

Additional details regarding experimental configurations, sample fabrication used in initial end-to-end infrastructure tests and algorithmic configurations are presented in the Methods section.

### Computational Architecture

#### Architecture Overview

From a high-level perspective, our approach follows a closed-loop architecture in which each newly acquired measurement initiates the next stage of the loop. Upon acquisition, raw detector images and associated metadata are registered to a data access service, where they can be accessed by downstream processing components. Subsequently, several data reduction steps are applied and relevant features are extracted. These features are then passed to a decision agent, which proposes subsequent measurement configurations and communicates these to the instrument control system. Intermediate results and their provenance (e.g., data origin and feature extraction parameters) are persisted and made available for downstream web-based visualization. The entire process forms an autonomous loop, illustrated schematically in Figure 1.

**Figure 1:**
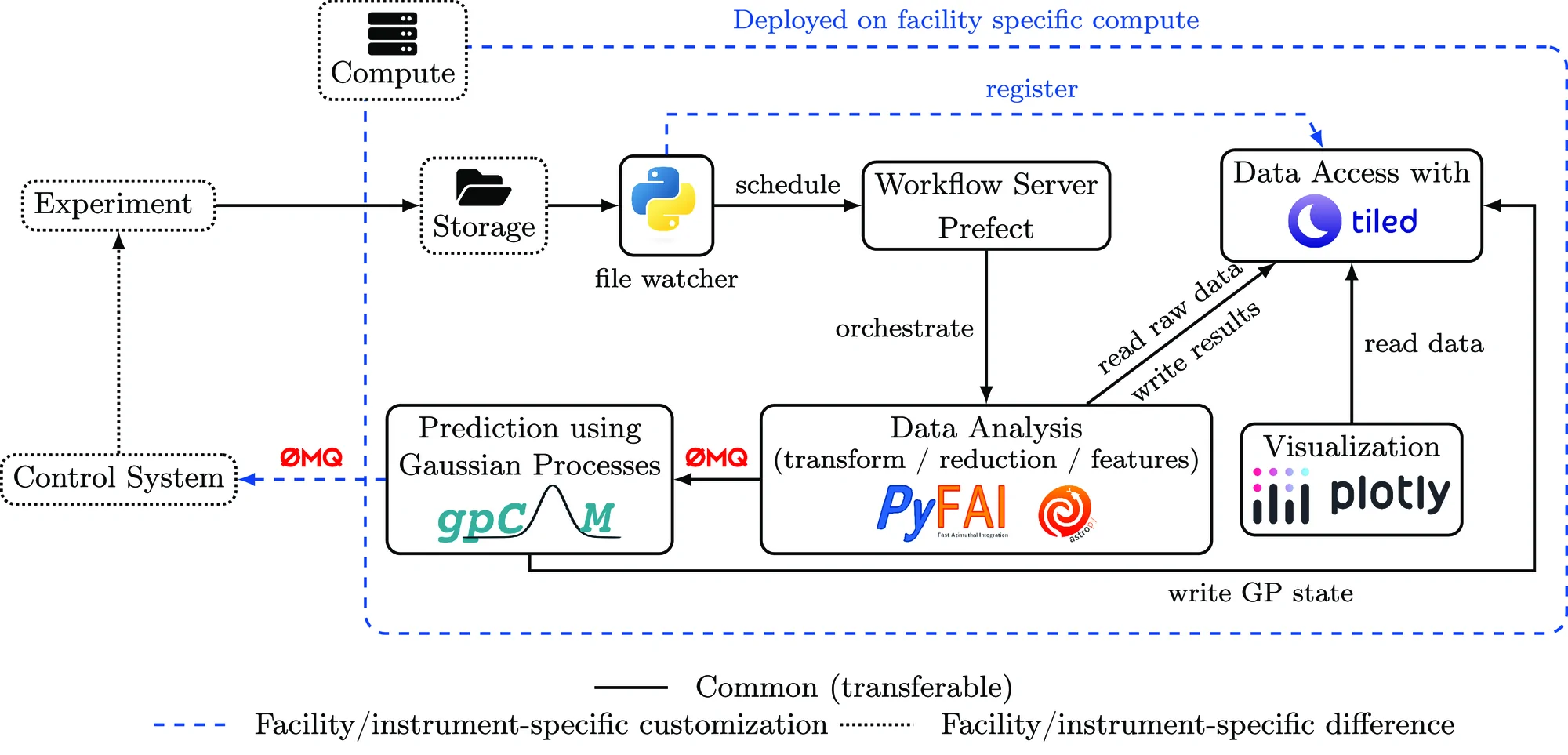
Schematic overview of the autonomous data processing pipeline. The closed-loop architecture integrates beamline control, data acquisition, data handling, reduction, feature extraction, and decision-making into a unified workflow.

#### Software Components

Our autonomous data processing pipeline builds on a set of established, Python-based open-source software frameworks that address data access, workflow management, visualization, optimization, feature extraction, and communication between distributed components.

The following key components comprise the system: **Prefect** (https://docs.prefect.io/) for workflow orchestration, **Tiled** (https://blueskyproject.io/tiled/) for unified data access, a web-interface based on **Plotly Dash** (https://dash.plotly.com/) for initial data reduction testing, **gpCAM** ^22^ (https://gpcam.lbl.gov/) for determining subsequent experiment steps through Gaussian process-based function approximation and uncertainty quantification, **pyFAI** ^23^ (https://pyfai.readthedocs.io/) for X-ray scattering specific data reduction steps, and **Astropy** ^24^ (https://docs.astropy.org/) for feature extraction. Communication between these components is facilitated through lightweight message bus systems such as **ZeroMQ** (https://zeromq.org/). Details on the respective software components and the purpose they serve are given below.

**Prefect** is a workflow orchestration platform enabling the combination of multiple Python-based data analysis steps into workflows and their execution in an optionally distributed manner. It has a web-based interface for scheduling, managing, and displaying the state of multi-step data analysis and machine learning pipelines. In our pipeline, it serves as the mechanism that unifies scheduling of feature extraction on diverse computing infrastructure.

**Tiled** is a data access service part of the Bluesky^25^ scientific Python ecosystem, which provides tools for experiment control, data acquisition, and data management at synchrotron and laboratory instruments. Tiled can access filesystems and databases to enable search and structured, sliceable access to many types of data structures such as N-dimensional strided arrays, sparse arrays, tabular data, nested variable-sized arrays, and hierarchical structures. Using Tiled allows us to build data analysis steps on these data structures rather than the specific file formats in use at a particular instrument. We additionally use Tiled to manage intermediate outputs and to retrieve data for downstream analysis and visualization. As a result, it represents the most tightly integrated component in the current implementation. Other components operate on in-memory data representations and can be exchanged more readily. This choice also provides a natural foundation for tighter integration with Bluesky-native execution and orchestration components as they become available at participating facilities.

**pyFAI** ^23^ is a library for fast azimuthal integration of X-ray scattering data. We make use of it for transformation of 2D detector images into 1D intensity profiles. In the first set of experiments, we focused on data reduction workflows in transmission geometry, but an extension to grazing-incidence geometry has since been realized.

**gpCAM** ^9,22^ is a Bayesian optimization and adaptive experimental design toolkit. It provides Gaussian process-based models for optimization and uncertainty quantification, enabling efficient decision-making and adaptive data acquisition.

**ZeroMQ** is a messaging library that provides various mechanisms for inter-process communication. It is used in our pipeline to facilitate communication between distributed components, such as sending motor positions from the decision agent to the beamline control system and streaming features back.

**Apache Kafka** (https://kafka.apache.org/) is a distributed event streaming platform. Kafka is used by the underlying storage infrastructure^26^ of the Maxwell Cluster at DESY to expose storage events, which can optionally be used to specifically trigger (distributed) actions on HPC nodes.

Overall, the specific software components used here represent one concrete realization of a modular autonomous loop architecture. Their selection was guided by practical criteria relevant to cross-facility deployment, including portability, active maintenance and community adoption, and clear interface boundaries between data access, workflow orchestration, and decision-making. Several components were already in active use at participating facilities or in closely related projects. Within this design, alternative tools for workflow orchestration, Gaussian process modeling, messaging, or visualization can be substituted without altering the overall system structure.

The chosen software components and the modular nature of the architecture get us most of the way towards a facility-agnostic setup. However, as detailed in the following sections, some adaptations are necessary to integrate with existing hardware and software infrastructure at each facility. Table 1 summarizes these aspects.

**Table 1:** Summary of Facility-Specific Adaptations for Control Integration, Data Access (File I/O), and Computing Deployment, and the Software Packages Used to Unify the Autonomous Loop across facilities

category	adaptations ALS	adaptations PETRA III	unification
Controls integration	Adhere to message format of **BCS API**	Custom **Sardana** Macro	**ZMQ**
Data Access	Custom adapter for.edf,.gb, and meta data text files written by the PILATUS3 2M detector and the LabVIEW control system	Custom adapter for.cbf and groups of.nxs files written by the PILATUS 2M and the LAMBDA 9M detectors	**Tiled**
Computing Hardware	Deployment on local data analysis machine	Deployment on Maxwell utilizing SLURM for scheduling	**Prefect**

#### Controls Integration

The integration of beamline-specific control and data acquisition into an autonomous loop requires programmatic access to change measurement configurations and trigger new acquisitions. At ALS Beamline 7.3.3, this access is provided through a LabVIEW-based system with the ALS-developed **BCS API**, which assembles messages according to the API’s predefined structure. At PETRA III P03, by contrast, which provides controls through **Tango** and **Sardana**,^27,28^ we implemented a custom Sardana routine that includes a **ZeroMQ** listener, giving us flexibility to define the message format. Despite these differences, both setups enable a common communication pattern where commands are defined by type (e.g., a motor move) with associated metadata such as motor names and target positions, forming a lightweight but extensible protocol. Acquisition parameters such as exposure time and detector configuration are handled through existing beamline configuration and are not modified through the messaging layer.

#### Data Access

Because both facilities deliver detector files in facility-specific formats with additional metadata recorded in text logs, we use custom adapters to ingest data into Tiled. These adapters load detector frames (e.g.,.edf,.cbf,.gb), merge them with metadata from file headers and associated log files, and expose them as uniform array-plus-metadata structures. This ensures that downstream reduction and decision steps operate consistently across facilities, independent of file formats or logging conventions.

#### Data Reduction and Feature Extraction

We implemented a reduction workflow that performs azimuthal integration of two-dimensional detector images into one-dimensional intensity profiles, a standard approach for SAXS/WAXS data analysis. The workflow uses **pyFAI** ^23^ for integration and **astropy** ^24^ for fitting a pre-defined number of peaks as a sum of Gaussian or Voigt functions to extract key parameters such as peak position and width.

Because the reduction pipeline forms an integral part of the autonomous experiment, we developed a Dash-based interface to visualize intermediate results and validate reduction parameters (Figure 2). The interface accesses data through Tiled and provides users with tools to select detector images, masks, and set calibration parameters, and to inspect the resulting integrated profiles. This view is directly orchestrated through the same Prefect-based infrastructure used for the autonomous loop, ensuring that the data-reduction workflow can be validated and executed reliably within the overall system. Individual steps such as loading data and masks, performing the integration, filtering invalid values, and fitting peaks are implemented as separate Prefect tasks, meaning each operation runs as an independent, orchestrated unit that can be reused or cached.

**Figure 2:**
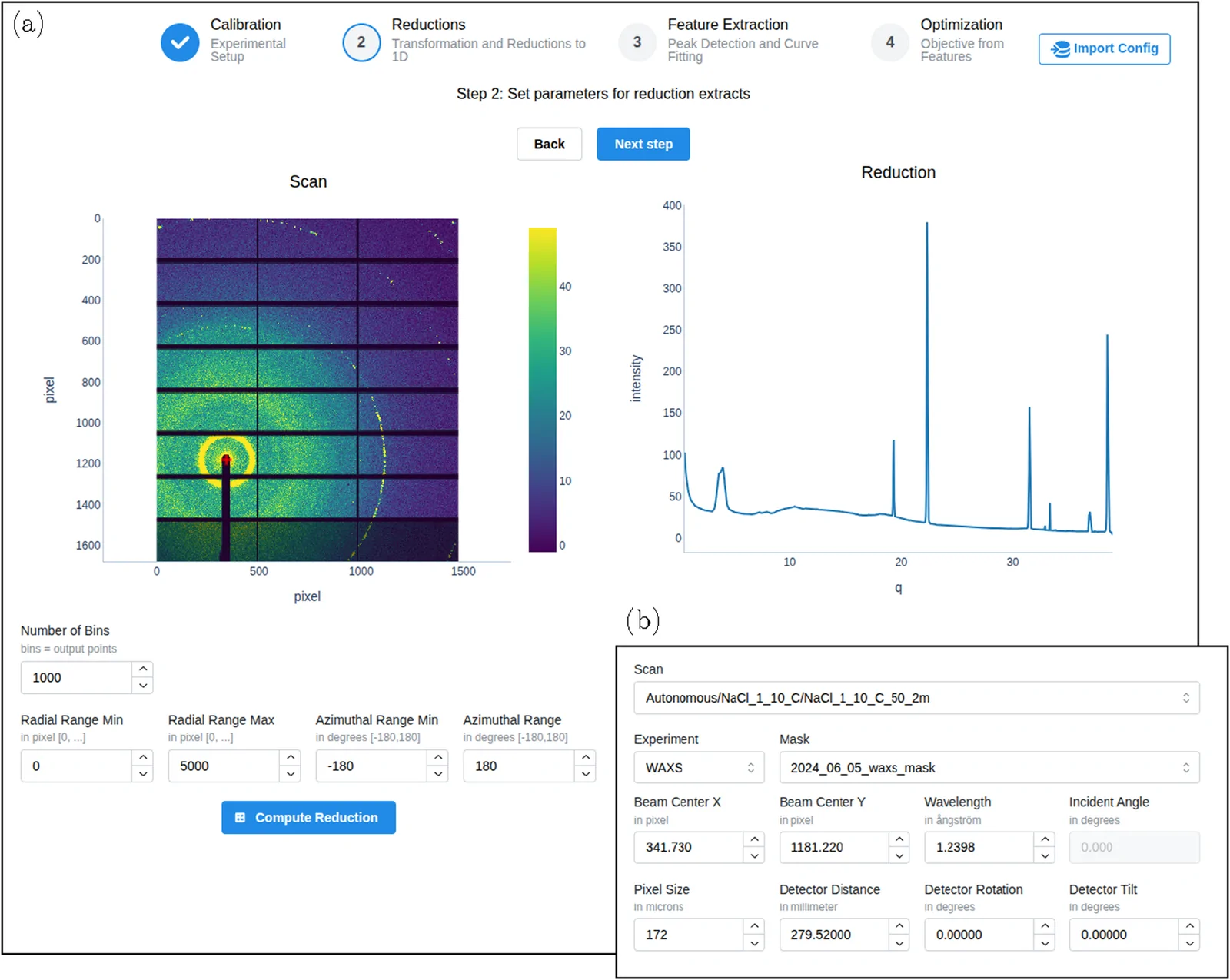
Dash-based user interface for testing and validating the results of data reduction workflows. (a) Shows the state of the interface after the user has selected a raw detector image and a suitable mask and applied azimuthal integration as the default reduction for WAXS under the second step. The inset (b) shows the calibration parameters that need to be provided in the first step of the workflow.

We envision this interface to also support configuration of subsequent steps, such as feature extraction and Gaussian-process setup, but these components were not yet implemented at the time of the experiments presented here.

#### Computing Hardware

At ALS beamline 7.3.3, data reduction and decision making were carried out on a dedicated analysis machine located adjacent to the beamline data acquisition computer. This setup enabled low-latency feedback during experiments without depending on central compute resources. In contrast, at PETRA III beamline P03, reductions and decision tasks were executed on the Maxwell cluster, the central computing infrastructure at DESY. Prefect orchestration allowed the same workflows to be scheduled on either local machines or central resources, demonstrating that the approach can flexibly target different computing environments without modifying the core autonomous loop.

#### Autonomous Decisions

The features extracted during data reduction, such as peak positions or intensities, serve as regression targets for the Gaussian-process (GP) decision model implemented through **gpCAM**.^22^ gpCAM operates in an iterative loop, in which each new measurement is analyzed and incorporated into the GP model to inform subsequent acquisitions. We make use of gpCAM’s default anisotropic Matérn kernel, which allows different characteristic length scales along the *x* and *y* motor coordinates and assumes that correlations depend only on relative distance.

Sampling points are selected using a variance-based acquisition strategy that targets regions of high model uncertainty to most efficiently improve the surrogate. The selected coordinates are communicated to the beamline through ZeroMQ, and the resulting measurements are passed through the same reduction and feature-extraction pipeline before being fed back to the model. Posterior mean maps and information about how they were derived are periodically written to Tiled, providing a continuously updated view of the model during the experiment. Together, these components close the loop between measurement, analysis, and decision making, allowing the system to refine its sampling strategy in real time.

### Case Studies

The autonomous workflow is tested under real experimental conditions at two synchrotron beamlines to evaluate its performance and transferability. These case studies demonstrate how the same infrastructure operates across distinct environments with different control systems, detectors, and sample types.

We conducted end-to-end infrastructure tests at both the Advanced Light Source (ALS, beamline 7.3.3) and PETRA III (beamline P03) to validate the autonomous loop across two distinct facility environments. Our focus was on demonstrating that the integration of control, data access, feature extraction, and decision making can be realized in practice, and that the resulting system can successfully explore samples with spatially varying scattering characteristics. For our initial tests, we selected materials with reproducible and high-contrast scattering signatures, using sample mapping as a common but intentionally straightforward proof-of-concept task. The configurations used at each facility are summarized in Table 2, highlighting differences in beamline setup and Gaussian-process parameters while applying the same acquisition strategy. Overviews of each experiment are shown in Figures 4 and 5.

**Table 2:** Measurement and Acquisition Configurations for Autonomous Scattering Experiments Performed at ALS 7.3.3 and PETRA III P03^a^

category	ALS (beamline 7.3.3)	PETRA III (Beamline P03)
Beam energy	10 keV	12 keV
Detector setup	PILATUS3 2M (WAXS, Sample-detector distance (SDD) = 279 mm)	PILATUS 2M (SAXS, SDD = 9270 mm) + LAMBDA 9M (WAXS, SDD = 340 mm)
Sample type	NaCl solution dried on Kapton tape exhibiting coffee-ring effect	Co-extruded composite with aluminum ring and PA6 polymer core
Initial acquisition points	25 random points	5 random points
Motor ranges	*X* ∈ [8, 22] mm, *Y* ∈ [12, 26] mm	*X* ∈ [−10, 8] mm, *Y* ∈ [−8, 8] mm
Surrogate model output	*q*-space position of highest intensity peak	Beamstop diode value as indicator of degree of absorption
Acquisition strategy	Variance	Variance
GP kernel	anisotropic stationary Matérn kernel	anisotropic stationary Matérn kernel
Hyperparameter bounds	*σ* ^2^ ∈ [0.0001, 10^4^], *l*_*x*_,*l*_*y*_ ∈ [0.01, 100]	*σ* ^2^ ∈ [10^4^, 10^9^], *l*_*x*_ ∈ [0.05, 25], *l*_*y*_ ∈ [0.05, 9]
Initial hyperparameters	*σ* ^2^ = 1.272, *l*_*x*_ = 1.0, *l*_*y*_ = 3.745	*σ* ^2^ = 10^4^, *l*_*x*_ = 25, *l*_*y*_ = 8.999
Final hyperparameters	*σ* ^2^ = 1.249, *l*_*x*_ = 0.7027, *l*_*y*_ = 2.144	*σ* ^2^ = 3.691 × 10^8^, *l*_*x*_ = 1.158, *l*_*y*_ = 1.12
GP training interval	every 25 data points	every 50 data points
Runtime	≈27.5 min	≈ 5.5 h
Number of data points	99	1000

a The table lists beam, detector, and sample parameters, along with the settings used for Gaussian-process modeling and acquisition during each experiment.

At ALS beamline 7.3.3, the autonomous loop was applied to a test sample containing regions with and without salt deposits. Scattering patterns from these areas differed in the position of their dominant peaks, reflecting the coffee-ring effect from dried sodium chloride on Kapton tape. Two representative measurements are shown in Figure 3: regions with salt deposits exhibit peaks at higher *q* values, while Kapton-only regions are dominated by the substrate peak. The position of the most intense peak was used as the surrogate model output for the Gaussian-process (GP) regression, which guided motor movements to sample areas of highest uncertainty. As more data points were collected and the GP was periodically retrained, the characteristic length scales of the anisotropic Matérn kernel decreased, indicating that the model adapted to sharper spatial transitions between regions with and without salt while still capturing variations within the salt-rich areas. The evolution of the GP mean over time is shown in Figure 4, where the sample representation becomes progressively refined. The workflow produced an adaptive overview of the sample in 27.5 min, compared to approximately 3 h 46 min for a conventional 0.25 mm raster scan that uniformly covered the same region.

**Figure 3:**
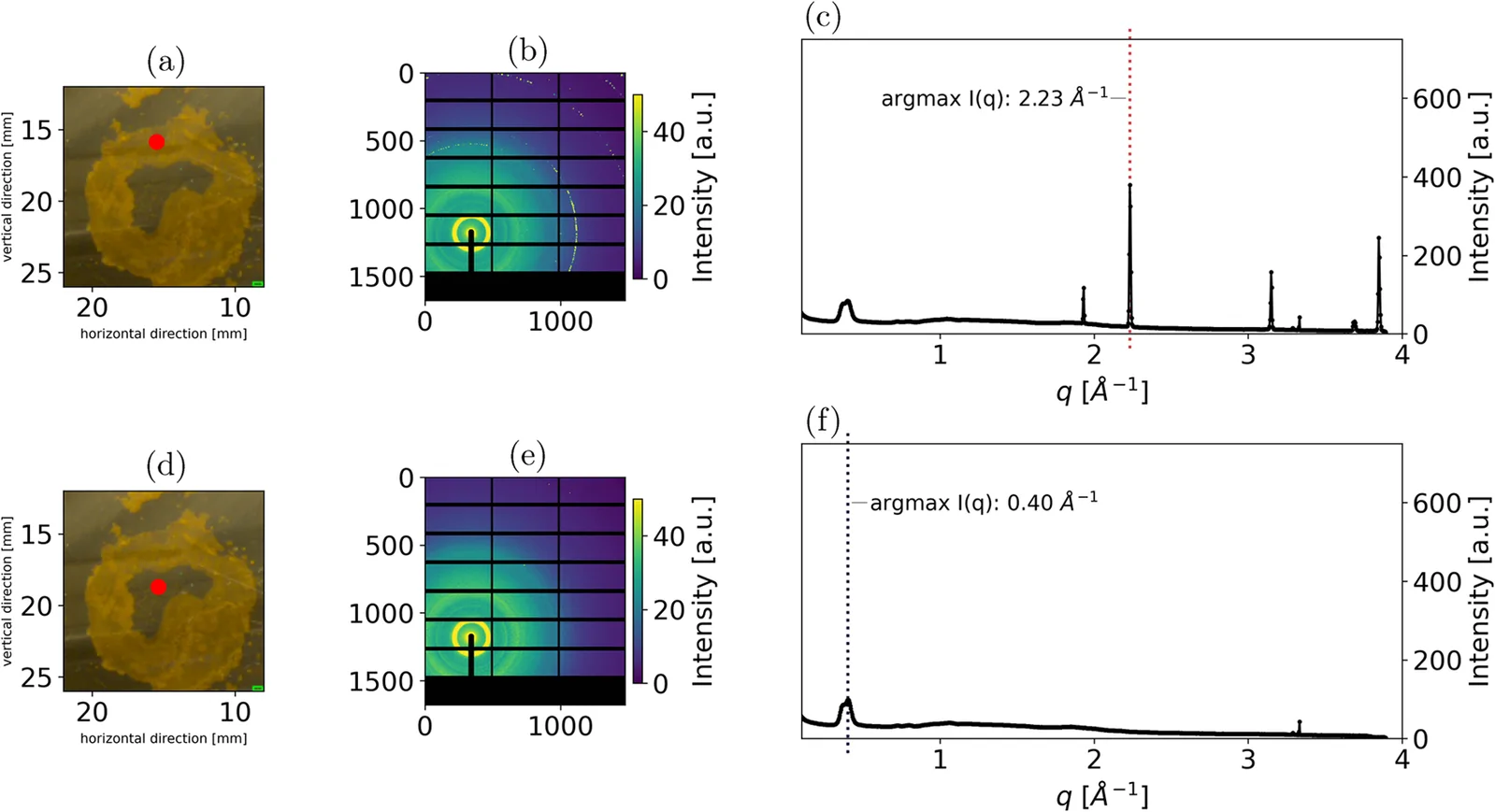
Scattering patterns acquired at locations with salt deposits exhibit peaks at higher *q* values than regions without salt, where the dominant peak corresponds to Kapton. Two representative measurement positions are shown in optical images taken along the beam direction, assembled in post-processing for visualization and not available to the autonomous decision-making during the experiment: with salt (a) and without salt (d). The corresponding raw detector images are shown in (b, e), and the reduced data with the extracted feature used in the autonomous loop are shown in (c, f).

**Figure 4:**
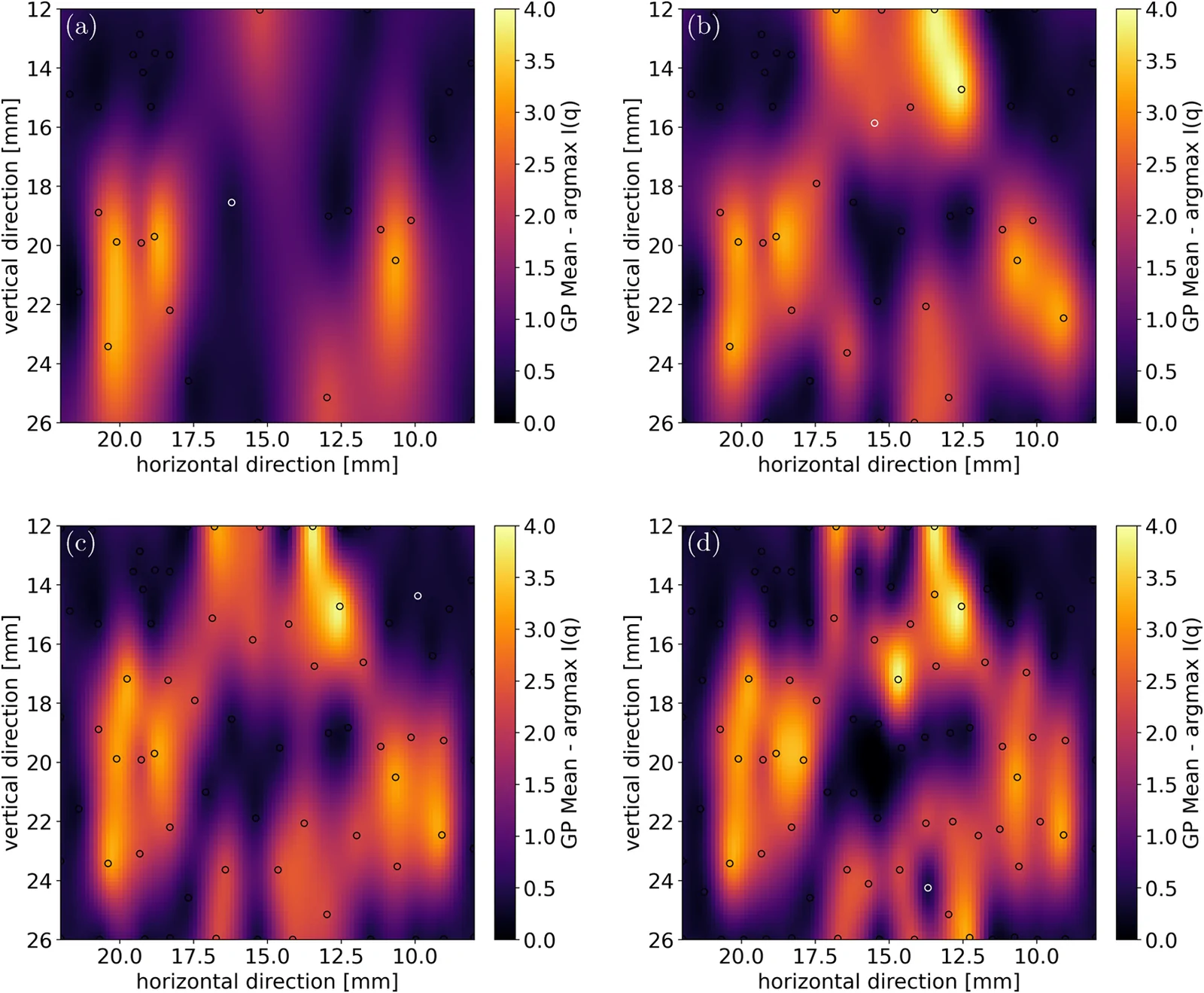
Overview of the autonomous mapping process for the sodium-chloride sample at ALS. (a–d) show Gaussian process means after *N* = 30, 50, 75, and 99 measurements, respectively.

At PETRA III beamline P03, the same autonomous workflow was deployed with configuration adjustments limited to the local control and data environment. The sample, a co-extruded aluminum/polyamide (PA6) composite, presented a sharply contrasting structure, with strong absorption from the aluminum ring and transmission through the polymer core. The beamstop diode signal served as the surrogate model output for the Gaussian-process (GP) regression, which directed measurements toward regions of highest predicted uncertainty. For this experiment, the GP hyperparameter ranges were defined to better match the physical scale of the mapped area, helping to avoid unrealistically large length scales while still allowing the model to adapt during optimization. As data accumulated and the GP was periodically retrained, the characteristic length scales of the anisotropic Matérn kernel decreased, reflecting a progressively finer representation of the spatial variation across the entire sample. The evolution of the GP mean throughout the experiment is shown in Figure 5, where the model incrementally refines its overall understanding of the measurement space. The complete experiment ran for approximately 5.5 h, using the same orchestration components on the Maxwell cluster while interfacing with the Sardana control system. Note that the higher overall runtime is primarily due to the number of measurements in the experiment.

**Figure 5:**
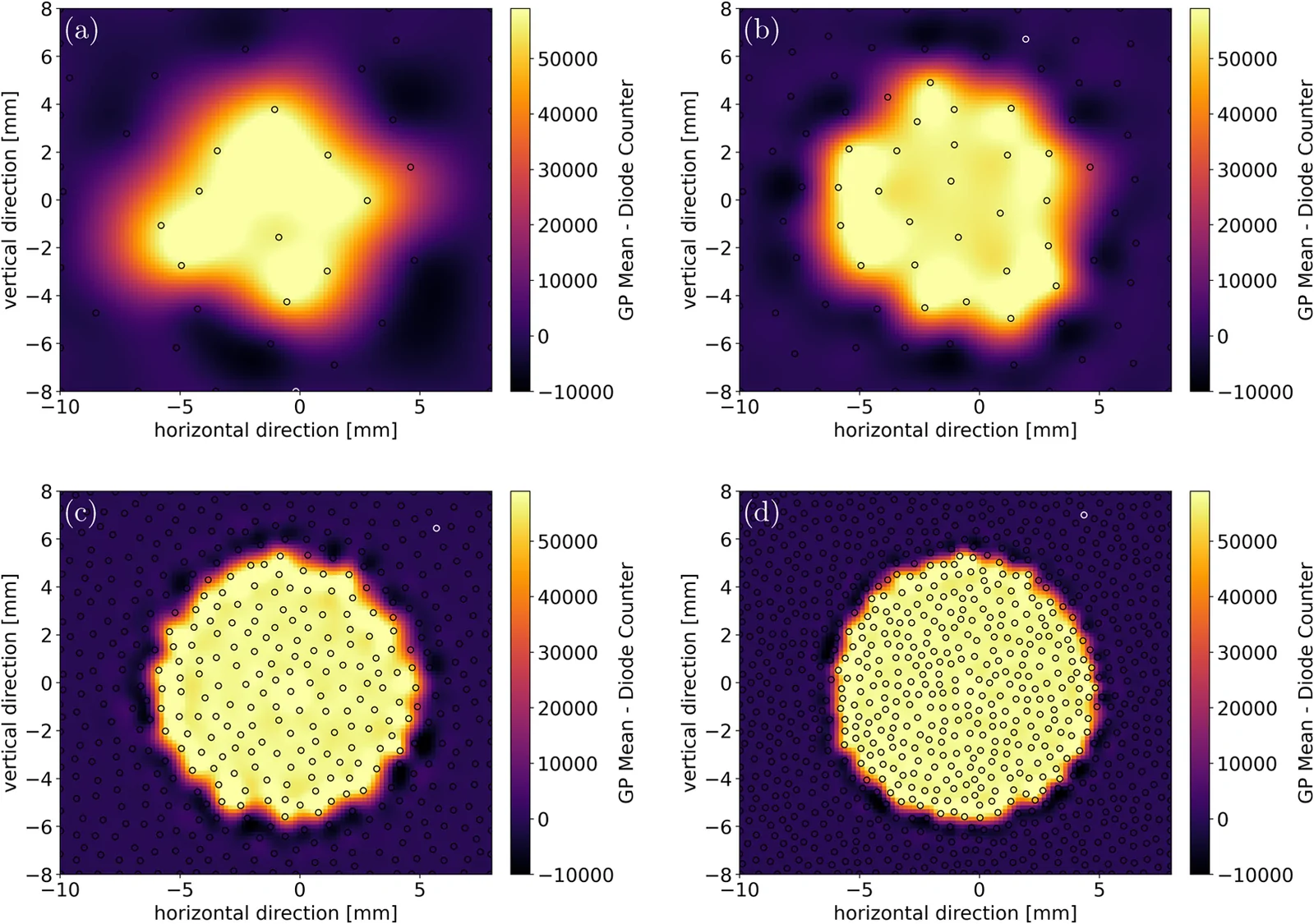
Overview of the autonomous mapping process for the composite sample at PETRA III. Panels (a–d) show the Gaussian process mean after *N* = 50, 100, 500, and 1000 measurements, respectively.

Across both facilities, the Gaussian-process model exhibited similar behavior as it progressively refined its representation of the measurement space with each new data point and retraining. The small differences observed between experiments reflected the characteristics of the samples and the local experimental configurations rather than differences in how the model operated. Only thin adapters were required to handle facility-specific control and data formats, while all higher-level components such as data ingestion, orchestration, and decision making remained identical. These results demonstrate that the workflow is readily transferable across beamlines and maintains comparable performance and convergence behavior in distinct experimental environments.

## Discussion

The consistent performance of the autonomous loop across facilities highlights the robustness of a modular, message-based design that can operate across different beamline environments. This cross-facility demonstration reinforces the practicality of modular approaches for integrating autonomous workflows into existing control systems and detectors. Building on this foundation, our next steps will focus on two complementary priorities: expanding the scientific scope and improving system responsiveness to support future autonomous operation across facilities.

Scientifically, our next step is to target problems where the extracted features carry direct physical meaning–for example, morphology in polymers and composites where optimization of these features, rather than their spatial mapping, is of primary scientific interest. Beyond peak detection, this includes domain spacing and spread (peak position and FWHM), texture and anisotropy (sector integrations), and multi-peak or phase-fraction fits. The same interfaces used here allow us to substitute alternative feature extractors and to compare decision agents and acquisition rules (e.g., multi-objective formulations that balance signal quality with motion overhead). Importantly, the underlying optimization remains unchanged when substituting feature definitions, and the flexibility to encode prior knowledge through appropriate GP kernel choices enables sensitivity to a wide range of variations in these physically meaningful objectives. We also plan to explore learned representations as features, using compact embeddings from machine-learning models to guide exploration alongside engineered descriptors, supported by interactive tools such as **LatentSpace Explorer**.^29^

A related practical consideration is that autonomous experimentation shifts a significant portion of effort toward upfront configuration. In particular, the selection of features, objective functions, and reasonable parameter ranges requires domain expertise and careful validation. While the optimization loop itself operates generically once defined, these design choices strongly influence convergence behavior and scientific relevance. In the demonstrations presented here, such parameters were informed by prior experimental experience and expert guidance. Providing structured defaults, validation tools, and clearer guidance for non-expert users remains an important direction for future work as autonomous methods are adopted more broadly.

On the systems side, reducing latency and increasing throughput remain priorities. In our initial demonstrations, file-based processing introduced delays because analysis waited for files to land on central storage. We are incorporating Prefect’s task-level caching to avoid recomputing invariant steps (calibration, masks, geometry) and to reuse intermediate reductions when parameters are unchanged. In parallel, we are moving toward more event-driven execution with direct data streaming and lightweight notifications so new scans are acted on immediately. **Arroyopy** (https://github.com/als-computing/arroyopy) and **ASAP::O** (https://asapo.pages.desy.de/asapo/) are tools under development that could support this integration. We also identified some opportunities for parallelization and further decoupling of exporting intermediate results from processing steps. Additional gains could be achieved by incorporating motion-aware acquisition strategies, such as travel-cost–aware point selection or flyscan-style continuous acquisition, although such extensions introduce additional complexity beyond the scope of the present demonstrations. These priorities remain central as beamlines transition to modern control stacks geared toward programmatic remote control and streaming, an evolution accelerated by upcoming high-throughput beamlines such as those enabled by the ALS-U upgrade. The framework introduced here was designed with scalability and flexibility in mind, and we expect lower end-to-end latency and more robust autonomous operation across instruments as adoption of modern control and data-streaming infrastructures continue to mature.

As these infrastructures mature and additional data streams become accessible within the same event-driven framework, richer sources of experimental context can be incorporated into autonomous decision-making. In practice, human operators often accelerate exploratory measurements by leveraging real-space contextual information, such as visual feedback from cameras or other auxiliary diagnostics. Translating this capability to autonomous operation would require synchronized auxiliary data streams and calibrated mappings between contextual coordinates and scan positions. Context-derived region-of-interest masks or constrained space-filling strategies could provide informative initialization and guidance for Bayesian optimization, reducing search overhead while preserving the core decision-making framework. This capability is particularly relevant in multimodal experimental settings, where multiple complementary data streams provide additional experimental context. In such settings, multimodal machine-learning approaches are particularly well suited to encode heterogeneous inputs (e.g., scattering data, images, or metadata) into compact feature embeddings that serve as objectives or guide exploration, providing an alternative to analytically derived features. Additional context may also encompass externally defined metadata, such as sample fabrication parameters, experimental provenance, or information managed by facility sample-tracking or Laboratory Information Management Systems (LIMS), that can inform objective definition and define or constrain the explored parameter space without altering the underlying autonomous loop.

## Conclusion

We presented a modular workflow for autonomous scattering experiments and validated it at two distinct synchrotron beamlines. By separating facility-specific adapters (for controls and file ingestion) from shared components for data access, reduction, visualization, and decision making, we kept the core autonomous loop unchanged while accommodating different detectors, control systems, and compute environments.

Equally important, the process of adapting and deploying the workflow across facilities helped clarify the boundaries between local and general-purpose components. Local adapters for control and file ingestion could evolve independently, while shared orchestration, analysis, and decision layers remained stable. This modular separation improved maintainability by isolating local changes from global workflow dependencies.

A defining feature of the effort presented here was cross-collaboration among controls engineers, computing staff, beamline scientists, and materials scientists at both facilities. Through alternating tests at both sites and jointly defining analysis priorities and interface requirements, the teams were able to generalize the implementation and avoid specialized, tightly coupled solutions. The result was a framework that integrates smoothly with existing control environments yet remains adaptable for future extensions.

Because analysis operates on structured data rather than file formats, orchestration targets tasks rather than machines, and decision agents interact with message interfaces rather than instrument details, the workflow is inherently transferable. The same design principles that enabled replication across beamlines also support incremental development: new analysis tools or optimization algorithms can be introduced without altering the overall architecture. This flexibility will be essential as autonomous methods expand to a wider range of experimental goals and beamline configurations across the light-source community.

## Methods

### Measurement Configuration

To demonstrate the infrastructure, we conducted autonomous SAXS/WAXS experiments at ALS (Beamline 7.3.3) and PETRA III (Beamline P03). The following subsections provide a brief overview of sample characterization with SAXS/WAXS in general, as well as specific beamline configurations.

#### Small- and Wide-Angle X-ray Scattering

Small- and Wide-angle X-ray scattering (SAXS/WAXS) are non-destructive techniques to study structural properties in material science. By combining both techniques, structural features, e.g., grain sizes and particle shapes, domain walls, macromolecular arrangements in solution and thin films, as well as crystallite sizes can be obtained, ranging from several micrometers down to a few Ångströms. Spanning several orders of magnitude in spatial resolution, SAXS nicely bridges the gap between optical and electron microscopy and, especially in solutions, enables morphological studies without harsh sample manipulation, i.e., freeze drying. In a schematic scattering experiment (see Figure 6), the incoming X-rays can be described as a wave front denoted by its wave vector *k⃗*_*i*_ in reciprocal space. Upon interaction with the sample, the electrons scatter the incoming X-rays, and the resulting scattered wavefront is denoted by its wave vector *k⃗*_*f*_. The scattering condition

1 $$\mathrm{q⃗}=\vec{k_{f}}-\vec{k_{i}}$$

yields, for elastic scattering (|*k⃗*_*i*_| = |*k⃗*_*f*_|), the so-called scattering vector

2 $$q=\frac{4\pi }{\lambda }\sin\theta$$

with

3 $$2\theta =\arctan\frac{∥\left(X-X_{0}\right)\cdot \Delta_{p}∥}{\mathrm{SDD}}$$

where *λ* describes the X-ray wavelength, *X* the 2D coordinate on the detector, *X*_0_ the beam center position in 2D, Δ_*p*_ the pixel size, and SDD the sample-to-detector distance. Together, eqs 1–3 define the scattering vector in reciprocal space and relate it to the detector geometry. In a scattering experiment, the scattered X-rays are collected on large-area detectors, and the resulting intensity distribution over *q* is achieved by azimuthally integrating the obtained 2D images. Here, software packages like pyFAI^23^ offer standard routines for this integration process, beam center calibration, masking, as well as polarization and geometry corrections. From the resulting intensity profiles shape and size information (SAXS) as well as molecular and atomic structures (WAXS) can be extracted.

**Figure 6:**
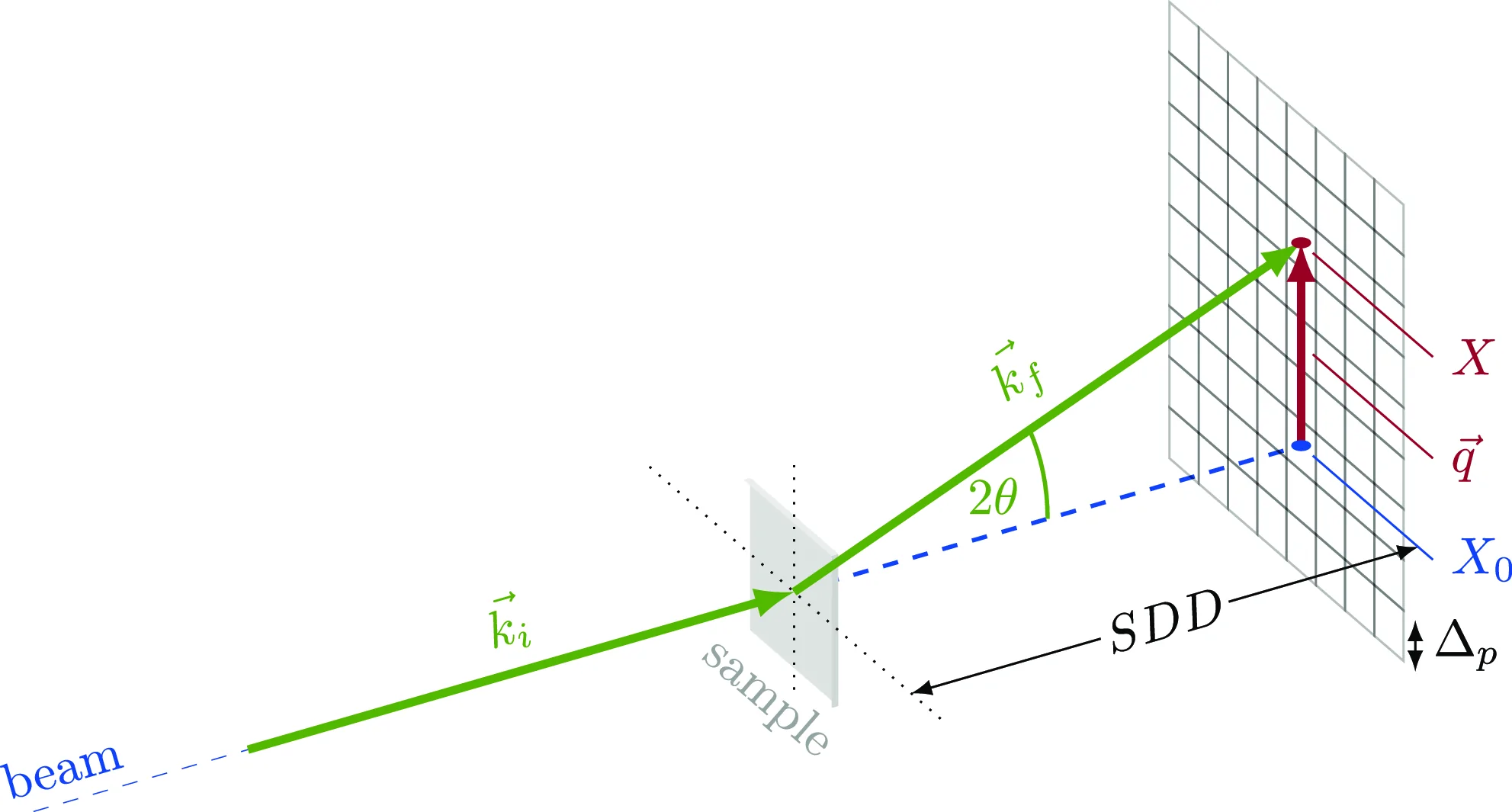
Schematic of a small-angle scattering experiment in transmission geometry. The incident beam $\vec{k_{i}}$ passes through the sample and is scattered by an angle 2*θ* toward the detector placed at a sample-detector distance *SDD*. The scattered beam $\vec{k_{f}}$ intersects the detector at pixel position *X*, offset from the beam center *X*_0_ by the momentum transfer vector *q⃗*. The detector pixel size is denoted by Δ_*p*_.

#### Beamline 7.3.3 at the Advanced Light Source

At the Advanced Light Source (Lawrence Berkeley National Lab (LBNL), Berkeley, California), beamline 7.3.3 offers hard X-ray radiation with a fixed energy of 10 keV (λ = 1.2398Å).^19^ For the experiments, a PILATUS3 2M detector (pixel size = 172 μm, Dectris, Ltd., Switzerland) was used, which was positioned 279 ± 1 mm away from the sample, and the beam size was approximately 300 × 700 μm^2^.

#### Beamline P03 at PETRA III

Beamline P03 is an experiment endstation at the synchrotron radiation source PETRA III (Deutsches Elektronen-Synchrotron (DESY), Hamburg, Germany), which specializes in surface and material characterization using SAXS and WAXS.^20^ Here, a PILATUS 2M (pixel size = 172 μm, Dectris, Ltd., Switzerland) detector and a LAMBDA 9M (pixel size = 55 μm, X-Spectrum, Hamburg) detector are used for data acquisition in SAXS/WAXS experiments, respectively. Both techniques were used simultaneously. For the present study, the X-ray energy was fixed at 12 keV (λ = 1.033 Å), and the X-ray beam was focused down to approximately 30 × 30 μm^2^. The two detector systems were positioned at 9270 ± 1 mm and 340 ± 1 mm for the SAXS and WAXS, respectively.

### Samples

A typical use case of autonomous experiments is to map a sample with spatially varying properties, either to identify regions of interest for further study or to assess sample composition and homogeneity. To demonstrate our autonomous infrastructure, we thus selected samples with spatially varying features. For our first validation tests, we focused on samples that can be distinguished based on simple features, and are relatively homogeneous. Figure 7 shows photographs of the samples used at each facility.

**Figure 7:**
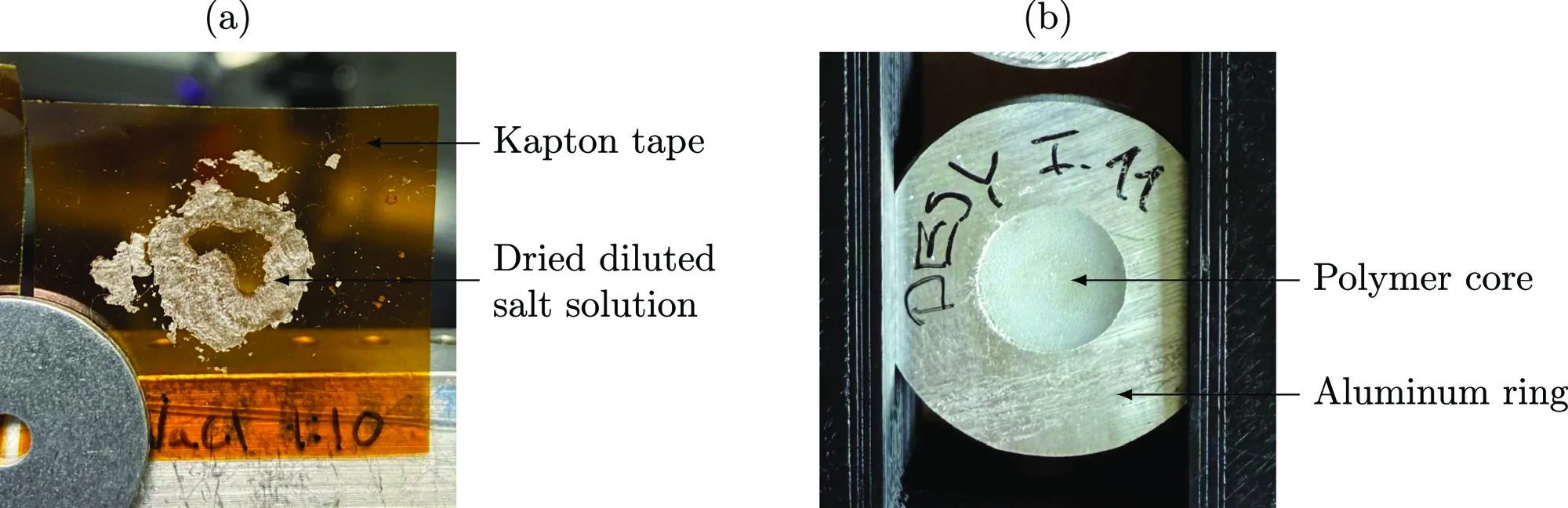
Photographs of samples mapped autonomously at the ALS (a) and PETRA III (b). In both cases, each spatial position can be assigned to one of two classes: salt/kapton (ALS) and aluminum/polymer (PETRA III).

At the ALS, we prepared samples by depositing droplets of sodium chloride solutions at two concentrations (e.g., 1 mg NaCl per 10 ml water and 1 mg NaCl per 20 ml water) onto Kapton tape. As the droplets dry, they exhibit the coffee-ring effect: evaporation occurs more quickly at the droplet edges than in the center, driving dissolved salt outward, causing interesting patterns in the final dried sample (Figure 7(a)).^30^ Sodium chloride is well-suited as a material to explore because it scatters X-rays strongly and produces distinct diffraction peaks. We use drop casting as it involves the fundamental process of droplet drying and evaporation-induced self-assembly, which is the basis for many fabrication processes in material science.^31–34^ Here, we build on our previous work on solution casting, where transmission SAXS (for thick coatings of colloids and hybrid materials) and GISAXS (for thin films and nanometer-sized colloids) were used to manually identify the different nanostructures induced by solvent evaporation.^30,35,36^

At PETRA III, we used a composite material comprising a disk-shaped, 2 mm thick aluminum profile cross section with a polymer core produced by an advanced co-extrusion process (Figure 7(b)). Specifically, as polymer basis the well-described polyamide type, *PA6 Durethan B31SK by Envalior, Düsseldorf, Germany*, has been investigated due to its possibility to form different process-induced structural features, i.e., crystalline phases. The samples were produced by intrinsic combination of aluminum extrusion with injection of extruded polymer melt into the welding chamber of a modified porthole die, whereby the polymer acts as a forming medium and solidifies in complex interactions with the moving aluminum shell - the processing die shapes the outer geometry, while the melt pressure defines the inner contour. Details of the sample processing can be found in Korkolis et al.^37^ The effects of coupled flow and solidification fields motivate the SAXS/WAXS analysis to identify local molecular orientations and to better understand the process-induced structure formation. Within the applied energy range, aluminum is largely absorbing, whereas the polymer core is comparatively transparent, yielding two distinct scattering regimes that are straightforward to classify. In a non-autonomous context, such samples are typically characterized with a linear scan across a sample cross-section radius, to locally map molecular orientations and process-induced polymorphic crystalline structures.

### Computational Architecture

#### Controls Integration

Programmatic access to configure newly suggested measurements was enabled through different control systems but unified via ZeroMQ-based messaging.

##### ALS Beamline 7.3.3

At Beamline 7.3.3, data acquisition is controlled by a LabVIEW-based system that coordinates the detector, motors, and optionally settings of custom sample environments. The system can be configured to accept remote commands via ZeroMQ, enabling programmatic access to motor positions and data acquisition. A Python client interface (called **BCS API**) provides high-level functions for motor movement and triggering of acquisitions, which we used in our experiments to autonomously coordinate the X and Y sample stage and initiate scans without manual user intervention.

##### PETRA III Beamline P03

Experimental control of the beamline end stations at PETRA III is based on **Tango** ^27^ and **Sardana** ^28^ for both hardware integration and delivering scripting capabilities. The latter relates to close-to-hardware control, while higher-level control and data acquisition is provided by Sardana. Sardana provides a set of pre-defined so-called scan macros to enable common scan types (e.g., grid scans over multiple inputs expressed through either absolute or relative positions), but also can be used to script complex sequences of motor movements and data acquisitions. This includes the ability to wait for specific conditions or events before proceeding with the next steps in the experiment.

To enable autonomous operation, we implemented a custom Sardana macro. This macro awaits movement commands, moves to a newly received motor position, executes single-shot acquisitions, streams counters via ZeroMQ, and then waits for updated motor positions from the decision agent. The macro is launched from **Spock** ^38^ (https://www.sardana-controls.org/users/spock.html), an IPython-based interface to Sardana, and allows the feedback loop to operate continuously without user intervention.

#### Data Access

edf and cbf are file formats written by the PILATUS3 2M and the PILATUS 2M detectors at ALS 7.3.3 and PETRA III P03 respectively. Both file formats consist of a text-based file header, followed by a binary chunk of data. Both are supported by the **fabIO** ^39^ (https://www.silx.org/doc/fabio/latest/) library that offers I/O capabilities for images produced by 2D X-ray detectors by various manufacturers.

At the ALS, data acquisition can be configured to acquire vertically offset detector images, which are then stitched together to eliminate horizontal detector gaps. The stitched result is made available as a.gb (General Binary) file, which can easily be understood as an array. For the associated meta data, header information and text logs of the non-stitched measurements are merged.

The LAMBDA 9M detector at P03 writes a collection of 11 NeXus files, one for each of the 11 detector modules. For visualization purposes, we assemble these into the full detector image making use of **h5Py** ^40^ (https://docs.h5py.org/).

#### Data Reduction and Feature Extraction

For the wide-angle X-ray scattering setup at ALS beamline 7.3.3, we determined the sample-detector distance and beam center coordinates using the **Irena** ^41^ (https://usaxs.xray.aps.anl.gov/software/irena) package within **Igor Pro**. A silver behenate (AgBh) calibration scan was used to refine the geometry parameters, yielding the following values: beam center (341.73, 1181.22) in pixel coordinates and a sample-detector distance of 279.52 mm. These parameters were subsequently applied in the azimuthal integration step of the data reduction workflow. An example of the resulting reduced profiles and extracted peak features is shown in Figure 3.

At P03, DPDAK^42^ was used to determine the detector geometry. Derived parameters were used solely to accurately describe the experimental setup in Table 2 (no azimuthal integration was performed). A unified, web-based tool for calibration is being developed to harmonize this step across facilities, although it was not used in the present experiments.

#### Autonomous Decisions

For the Gaussian-process surrogate, we use gpCAM’s default anisotropic Matérn kernel. Anisotropy is introduced through the distance metric *r* defined in eq 4 on the two motor coordinates

4 $$r\left(\left(x,y\right),\left(x^{\prime },y^{\prime }\right)\right)=\sqrt{{\left(\frac{x-x^{\prime }}{l_{x}}\right)}^{2}+{\left(\frac{y-y^{\prime }}{l_{y}}\right)}^{2}}$$

where *l*_*x*_ and *l*_*y*_ are the characteristic length scales along each axis. gpCAM employs the Matérn kernel^43^ with smoothness parameter ν = 3/2, given in eq 5

5 $$k\left(r\right)=\sigma^{2}\left(1+\sqrt{3}r\right)\exp\left(-\sqrt{3}r\right)$$

where σ^2^ denotes the signal variance. The surrogate’s hyperparameters σ^2^, *l*_*x*_, and *l*_*y*_ are initialized through optimization after acquisition at a number of random locations and then updated with the same optimization procedure at regular intervals during the autonomous experiment (every 25 data points at ALS and every 50 data points at PETRA III, see Table 2).

#### Computing Hardware

Data reduction, feature extraction, and autonomous decision-making were performed on two distinct computing platforms:

- At the ALS, all local analysis and workflow tests were executed on workstation near the beamline: A Mac Studio (2022) equipped with an Apple M1Max chip and 64 GB unified memory.
- At PETRA III, autonomous runs were executed on a single node of the Maxwell Cluster. The node features two AMD EPYC 7542 CPUs (128 cores total) and 512 GB RAM, connected via a high-speed Infiniband network to the facility’s shared storage system.

## Supplementary Material



## Data Availability

All experimental data supporting this study, including raw detector images, associated metadata text files, beamline-specific masks, reduced scattering profiles, and Gaussian-process model outputs, are publicly available via Dryad at 10.5061/dryad.p2ngf1w4t. The uploaded zip files should be extracted and organized in separate directories for ALS 7.3.3 and PETRA III P03, each containing subfolders for raw/ and processed/ data as detailed in the accompanying README. All code supporting data ingestion via Tiled, analysis and visualization, and configuration, as well as execution of the autonomous pipeline is openly available at https://github.com/illumine-project/saxswaxs-workflow-viz and https://github.com/illumine-project/saxswaxs-workflows.

## Notes

ALS-PETRAIII—autonomous scattering; animations of the two conducted experiments, measured data, associated features, and Gaussian process means per acquisition (MP4)
